# KEPPRA: Key Epilepsy Prognostic Parameters with Radiomics in Acute Subdural Hematoma Before Craniotomy

**DOI:** 10.3390/brainsci15020204

**Published:** 2025-02-16

**Authors:** Alexandru Guranda, Antonia Richter, Johannes Wach, Erdem Güresir, Martin Vychopen

**Affiliations:** Department of Neurosurgery, University Hospital Leipzig, 04103 Leipzig, Germany; antonia.richter@medizin.uni-leipzig.de (A.R.); johannes.wach@uniklinik-leipzig.de (J.W.); erdem.gueresir@medizin.uni-leipzig.de (E.G.); martin.vychopen@uniklinik-leipzig.de (M.V.)

**Keywords:** acute subdural hematoma, 3D Slicer, radiomic, epilepsy risk, craniotomy, elongation, generalized seizures

## Abstract

Background: Acute subdural hematoma (aSDH) is associated with a high risk of epilepsy, a complication linked to poor outcomes. Craniotomy is a known risk factor, with an epilepsy incidence of approximately 25%. This study evaluated radiomic features from preoperative CT scans to predict epilepsy risk in aSDH patients undergoing craniotomy. Methods: A retrospective analysis of 178 adult aSDH patients treated between 2016 and 2022 identified 64 patients meeting inclusion criteria. Radiomic features (e.g., Feret diameter, elongation, flatness, surface area, and volume) from preoperative CT scans within 24 h of surgery were analyzed alongside clinical factors, including cardiac comorbidities, pupillary response, SOFA score, age, and anticoagulation status. Results: Of the 64 patients, 18 (28%) developed generalized seizures. Univariate analysis showed significant associations with Feret diameter (*p* = 0.045), elongation (*p* = 0.005), cardiac comorbidities (*p* = 0.017), and SOFA score (*p* = 0.036). ROC analysis showed excellent discriminatory ability for elongation (AUC = 0.82). Multivariate analysis identified elongation as an independent predictor (*p* = 0.003); elongation ≥ 1.45 increased seizure risk 7.78-fold (OR = 7.778; 95% CI = 1.969–30.723). Conclusions: Radiomic features, particularly elongation, may help predict epilepsy risk in aSDH patients undergoing craniotomy. Prospective validation is needed.

## 1. Introduction

Acute subdural hematoma (aSDH) represents a critical condition in neurosurgery, often resulting from high-energy trauma such as motor vehicle accidents, falls, or violent impacts [1,2]. It accounts for a significant proportion of traumatic brain injuries (TBIs) worldwide, with an estimated incidence of 14 to 38 cases per 100.000 individuals annually [3]. According to already presented data, the mortality rates for aSDH may reach 40% to 60% [4,5,6]. Among survivors, long-term neurological deficits, cognitive impairments, and decreased functional independence are common, underscoring the severity of this condition [7].

Post-traumatic epilepsy (PTE) is one of the most challenging and debilitating complications following aSDH. Craniotomy, the standard surgical treatment for aSDH requiring evacuation [8], is itself associated with an increased risk of PTE [9]. Most studies report seizure incidence rates as high as 25% [10,11]. While the pathophysiology of PTE is complex, involving cortical disruption [12], inflammation [13], and neuronal hyperexcitability [14], the ability to predict which patients are at highest risk remains limited. Current predictive models focus on clinical variables like age, comorbidities, and injury severity. However, these factors often lack precision and do not reflect structural changes relevant to risk stratification.

Recent advances in medical imaging, particularly in the field of radiomics, offer a promising avenue for addressing this gap. Radiomics enables the extraction of high-dimensional, quantitative data from medical images, transforming conventional imaging into a robust source of parameters. In the context of acute brain injury, radiomic features such as geometry, texture, and spatial patterns may reveal insights into disease processes that are imperceptible to the human eye. Preoperative computed tomography (CT) scans, which are routinely performed in the acute management of aSDH, provide an opportunity to assess structural markers that may be predictive of PTE. Parameters such as Feret diameter, elongation, and semiautomatic volumetric data have shown potential in characterizing pathological changes associated with adverse outcomes, including epilepsy [15,16]. 

This study investigates the utility of radiomic features derived from preoperative CT scans in predicting generalized seizures in patients undergoing craniotomy for aSDH. By integrating radiomic parameters with clinical variables such as cardiac comorbidities, pupillary response, and SOFA score, this work aims to identify predictors of PTE. We hypothesize that specific imaging-derived features, particularly those reflecting structural deformation, may improve risk stratification. The findings of this study could provide a basis for personalized risk assessment, enabling clinicians to implement targeted monitoring and preventive strategies in patients at the highest risk. Through this approach, we hope to contribute to improved outcomes in the management of acute subdural hematoma. 

## 2. Materials and Methods

### 2.1. Study Design, Patient Selection, and Data Collection

This retrospective single-center study analyzed data from patients diagnosed with aSDH and treated at our institution between 2016 and 2022. The study protocol was approved by the Clinical Ethics Committee of the University of Leipzig (362/23-ek). Inclusion criteria were adult patients (≥18 years) who underwent craniotomy for aSDH evacuation and had preoperative CT scans performed within 24 h prior to surgery. The diagnosis of aSDH was confirmed by an interdisciplinary team comprising neurosurgeons and neuroradiologists. Patients with incomplete imaging data, insufficient clinical records, or a history of preexisting epilepsy were excluded from the analysis.

The occurrence of generalized seizures was diagnosed based on established clinical guidelines, including the International League Against Epilepsy (ILAE) classification criteria [17]. Generalized seizures were identified through clinical observation of tonic–clonic activity or in cases of uncertainty via electroencephalogram (EEG) monitoring. Focal seizures were classified based on clinical presentations such as localized motor or sensory symptoms, with or without impaired awareness, in accordance with ILAE standards. In ambiguous cases, EEG monitoring was also utilized to confirm the diagnosis of focal seizures and to exclude secondary generalization.

Documentation of seizure activity, including EEG findings for both generalized and focal seizures, was performed in collaboration with a multidisciplinary team, including attending anesthesiologists, neurosurgeons, and neurologists, to ensure diagnostic accuracy.

A total of 178 patients with a confirmed diagnosis of aSDH were initially identified. Sixty-four patients who underwent craniotomy met the eligibility criteria. Patients treated conservatively or with alternative surgical techniques, such as decompressive craniectomy, were excluded. Data on demographic variables, comorbidities, pupillary response, Sequential Organ Failure Assessment (SOFA) scores, and anticoagulation status were collected. Preoperative imaging data were analyzed to extract radiomic features, with a focus on quantitative parameters. All craniotomies were performed following a standardized surgical protocol, as described in our previous study [18].

### 2.2. Radiomic Feature Extraction

All 64 patients underwent a CT scan within 24 h after trauma. Additionally, only patients who underwent craniotomy within 24 h after the diagnostic CT scan were included in the study. All CT scans included had both 5 mm and 1 mm slice sequences; however, only the 1 mm sequences were selected for analysis. The imaging data were extracted in DICOM format and processed using 3D Slicer software (version 5.6.2).

Manual segmentation of the aSDH was performed independently by two authors to ensure accuracy and consistency. Following segmentation, a 3D model was generated for each aSDH. Radiomic features, including Feret diameter, flatness, surface area, elongation, volume, and sphericity, were then analyzed based on these models.

### 2.3. Statistical Analysis

Statistical analyses were conducted using IBM SPSS Statistics software (version 29.0, IBM Corp., Armonk, NY, USA). Univariate analysis was performed to explore potential associations between clinical and radiomic features and seizure outcomes. Continuous variables, such as age, SOFA score, elongation, sphericity, surface area, Feret diameter, and volume, were tested for normality using the Shapiro–Wilk test. Normally distributed variables were analyzed using independent *t*-tests, while non-normally distributed variables were evaluated using Mann–Whitney U tests. Categorical variables, such as gender, anticoagulation status, cardiac comorbidities, and pupillary response, were analyzed using Chi-square tests or Fisher’s exact test when cell counts were insufficient for Chi-square reliability.

The cut-off for the SOFA score was predefined as <5 based on prior literature [19]. Receiver operating characteristic (ROC) curve analysis was performed to evaluate the discriminatory ability of significant radiomic features. The area under the curve (AUC) was calculated, and optimal cut-off values for variables such as elongation (≥1.45) and Feret diameter (≥142.613 mm) were determined to dichotomize these predictors for further analysis.

Significant variables from the univariate analysis (*p* < 0.05) were included in a multivariate binary logistic regression model to determine their independent predictive value. Significant variables from the univariate analysis (*p* < 0.05) were included in a multivariate binary logistic regression model to determine their independent predictive value. We decided not to include cardiac comorbidities in the model, as they are already incorporated into the SOFA score, which could compromise the results of the multivariate analysis. Odds ratios (ORs) with 95% confidence intervals (CIs) were calculated for each variable. To account for multiple testing, a Bonferroni correction (α = 0.05/3 = 0.0167) was applied to adjust the significance threshold and reduce the risk of type I errors. Multicollinearity among predictors was assessed using the variance inflation factor (VIF) to ensure the validity of the model. Model fit was evaluated using the Hosmer–Lemeshow goodness-of-fit test, while the Nagelkerke R^2^ was reported to indicate the explained variance.

Results are presented as mean ± standard deviation (SD) for continuous variables and as frequencies and percentages for categorical variables. A *p*-value < 0.05 was considered statistically significant, with all tests conducted as two-tailed.

## 3. Results

### 3.1. Patient Characteristics

**Figure 1:**
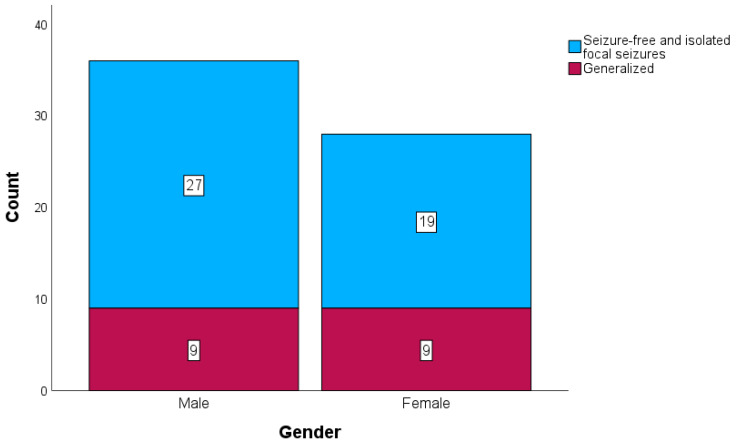
Distribution of epilepsy classification by gender.

The study cohort consisted of 64 patients who underwent craniotomy for acute subdural hematoma (aSDH). The mean age was 70.81 years (±16.10), with a range of 24 to 92 years. Of the patients, 36 (56.3%) were male, and 28 (43.8%) were female. Generalized seizures occurred in 18 patients (28.1%) following craniotomy, while the remaining 46 patients (71.9%) either experienced no seizures or had only isolated, self-limiting focal seizures. The distribution of epilepsy classification by gender is illustrated in Figure 1.

### 3.2. Univariate Analysis

The results of the statistical analyses are summarized in Table 1 and Table 2, offering a comprehensive overview of the associations between clinical and radiomic parameters and seizure classification.

Table 1 shows that cardiac comorbidities (*p* = 0.042) and SOFA score (*p* = 0.036) were significantly associated with generalized seizures.

**Table 1:** Univariate analysis of clinical and radiomic variables.

Variable	Overall Cohort (n = 64)	Seizure-Free/Isolated Focal Seizures (n = 46)	Generalized (n = 18)	*p*-Value
SOFA score	4.03 ± 2.96	4.53 ± 3.05	2.76 ± 2.30	0.036
Cardiac comorbidities	45 (Yes) vs. 19 (No)	29 (Yes) vs. 17 (No)	16 (Yes) vs. 2 (No)	0.042
Age	70.81 ± 16.10	68.85 ± 16.84	75.83 ± 13.09	0.119
Pupillary response	51 (Isocor) vs. 13 (Anisocor)	35 (Isocor) vs. 11 (Anisocor)	16 (Isocor) vs. 2 (Anisocor)	0.252
Gender	36 (m) vs. 28 (f)	27 (m) vs. 19 (f)	9 (m) vs. 9 (f)	0.583
Anticoagulation	33 (Yes) vs. 31 (No)	22 (Yes) vs. 24 (No)	11 (Yes) vs. 7 (No)	0.615

Table 2 demonstrates that among radiomic features, elongation (*p* = 0.005) and Feret diameter (*p* = 0.045) were significantly higher in patients with generalized seizures.

**Table 2:** Univariate analysis of radiomic variables.

Variable	Overall Cohort (n = 64)	Seizure-Free/Isolated Focal Seizures (n = 46)	Generalized (n = 18)	*p*-Value
Elongation	1.53 ± 0.29	1.47 ± 0.29	1.62 ± 0.19	0.005
Feret diameter (mm)	144.83 ± 24.93	140.90 ± 24.19	154.12 ± 20.20	0.045
Sphericity	0.27 ± 0.89	0.27 ± 0.79	0.27 ± 0.87	0.560
Surface area (mm^2^)	40,186.98 ± 21,796.96	39,624.77 ± 20,808.61	41,623.72 ± 24,728.10	0.744
Volume (cm^3^)	124.97 ± 244.24	133.62 ± 286.98	103.35 ± 60.74	0.796

### 3.3. ROC Analysis and Cut-Off Values

**Figure 2:**
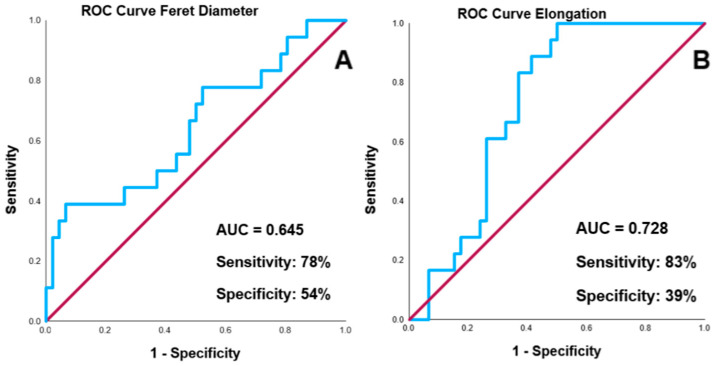
ROC curves for predicting generalized seizures: Feret diameter (**A**) and elongation (**B**).

To evaluate the predictive value of radiomic features for generalized seizures, ROC curve analysis was performed for elongation and Feret diameter (Figure 2). Elongation demonstrated good discriminatory ability with an AUC of 0.728, while the AUC for Feret diameter was 0.645.

### 3.4. Binary Logistic Regression

Binary logistic regression was conducted to evaluate the predictive value of clinical and radiomic variables for generalized seizures. The variables included in the model were dichotomized based on thresholds derived from the ROC analysis: elongation ≥ 1.45 and Feret diameter ≥ 142.613 mm. The SOFA score was dichotomized at a threshold of 5 based on criteria established in a previous study.

The logistic regression model identified elongation as a significant independent predictor of generalized seizures (OR: 7.778, 95% CI: 1.969–30.723, *p* = 0.003). Feret diameter showed an association with seizure risk (OR: 2.851, 95% CI: 0.853–9.515, *p* = 0.090), while the SOFA score did not reach statistical significance (*p* = 0.275). To adjust for multiple comparisons, a Bonferroni correction was applied (α = 0.05/3 = 0.0167). After this correction, elongation remained statistically significant, whereas the Feret diameter (*p* = 0.090) and the SOFA score (*p* = 0.275) did not reach significance. The overall model demonstrated moderate discriminatory ability, with a Nagelkerke R^2^ of 0.225 and a good fit indicated by the Hosmer–Lemeshow test (*p* = 0.909) (Table 3). 

## 4. Discussion

Our study highlights the potential utility of radiomic features derived from preoperative CT scans in predicting generalized seizures among patients undergoing craniotomy for aSDH. By focusing exclusively on preoperative data, we aimed to provide insights that could inform risk stratification prior to surgical intervention. Generalized seizures were observed in 28% of the cohort. Elongation emerged as an independent predictor of generalized seizures in multivariate analysis, with patients exhibiting elongation values above 1.45, demonstrating a 7.78-fold increased risk. Other parameters, such as Feret diameter and SOFA score, showed significant associations in univariate analysis, further emphasizing their relevance. However, only elongation retained significance in the multivariate model. These findings underscore the value of radiomic analysis as a preoperative risk assessment tool.

### 4.1. Craniotomy and Epilepsy in Managing Acute Subdural Hematomas

Acute subdural hematoma remains one of the most challenging acute conditions in neurosurgery, with high morbidity and mortality rates [20,21,22]. Prompt surgical intervention, most often craniotomy, is often essential to prevent life-threatening complications such as brain herniation and elevated intracranial pressure. However, while craniotomy can be a lifesaving procedure, it often exposes patients to additional complications, including a significant increase in epilepsy rates [11,23,24]. In our cohort, 28% of patients experienced generalized seizures, consistent with prior studies reporting seizure incidences of 25% to 30% [25,26].

Although critical for hematoma evacuation, craniotomy can exacerbate cortical disruption and trigger inflammatory processes, both recognized mechanisms in the development of epilepsy [27]. Surgical manipulation of brain tissue and exposure to air during the surgery may lead to neuronal hyperexcitability, further increasing seizure likelihood [28]. Additionally, while it remains a hypothesis, removing the hematoma could alter the cortical architecture in a way that creates new epileptogenic foci or reactivates dormant seizure-prone areas. This aligns with evidence linking structural brain injuries and surgical manipulation to changes in cortical function and increased seizure susceptibility [29,30]. The timing of the craniotomy is another crucial factor influencing patient outcomes. Early surgical intervention is associated with improved functional recovery but may also elevate the risk of immediate postoperative seizures due to rapid cortical decompression [31,32]. Conversely, delaying surgery might reduce short-term seizure risk but raise the possibility of secondary brain injury from prolonged intracranial hypertension. Incorporating radiomic features into preoperative evaluations could enable more nuanced risk assessments, optimizing surgical timing while considering individual seizure risks. Moreover, seizure prophylaxis, though not yet widely recommended, could be specifically targeted at patients identified as being at high risk of developing epilepsy, as previously reported by Sodal et al. [33]. 

Epilepsy in aSDH patients is further compounded by preexisting conditions, such as anticoagulation therapy and advanced age, both of which often influence the severity of hematoma and the outcomes of craniotomy [29]. In our study, anticoagulation showed limited direct predictive value for seizures. Similarly, while age significantly affects overall recovery and prognosis [34], it did not independently predict seizures in our cohort. Younger patients tend to benefit from greater neuroplasticity, which may mitigate cortical damage and lower the risk of seizures. In contrast, older patients often face diminished adaptability, making them more vulnerable to seizure development [35].

These findings underscore the importance of individualized approaches in aSDH management. While craniotomy remains the cornerstone of treatment, its inherent risks necessitate careful patient selection and strategies to minimize complications. Radiomic features, in particular, hold promise for providing valuable insights into individual seizure risks, allowing neurosurgeons to tailor perioperative care and improve outcomes for patients facing this critical condition. 

### 4.2. Anticoagulation, SOFA, and Comorbidities

The treatment of patients with aSDH undergoing craniotomy requires a nuanced approach, especially regarding the use of anticoagulation therapy. While anticoagulants are vital in reducing thromboembolic events, they are also linked to a higher risk of hematoma expansion and surgical challenges. Interestingly, in our cohort, anticoagulation was not found to be a significant predictor of seizure risk. This suggests that its primary influence may lie in exacerbating bleeding and hematoma severity, rather than directly affecting seizure mechanisms. Studies in other clinical settings, such as atrial fibrillation, have highlighted the potential for anticoagulants—including direct oral anticoagulants (DOACs) and vitamin K antagonists (VKAs)—to increase the risk of covert ischemic events. These events are established contributors to epilepsy and may be linked to subtle neuronal injury and inflammation [36]. Although these findings were not observed in the aSDH population, they underscore mechanisms by which anticoagulation could indirectly influence seizure susceptibility, particularly in patients with additional comorbidities.

Systemic inflammation appears to be an important factor linking elevated SOFA scores to an increased risk of generalized seizures, as identified in our analysis (*p* = 0.036). While the SOFA score primarily reflects organ dysfunction, higher scores often correlate with systemic inflammatory responses, especially in critically ill patients. Inflammatory processes can compromise the blood–brain barrier (BBB), enabling pro-inflammatory cytokines and other mediators to enter the central nervous system (CNS) and promote neuronal hyperexcitability [37]. Experimental studies have shown that markers such as CCL2 become upregulated in response to systemic inflammation, increasing seizure susceptibility [38]. The combination of BBB disruption and elevated cytokine activity within the CNS likely amplifies neuronal sensitivity, creating conditions conducive to seizures.

In addition, our analysis found a significant association between preexisting cardiac comorbidities and generalized seizures (*p* = 0.042). While these conditions are not inherently epileptogenic, they may heighten seizure risk through indirect pathways. Cardiovascular diseases are frequently accompanied by systemic inflammatory responses, which can disrupt the BBB and enhance neuronal hyperexcitability, thereby lowering the seizure threshold [37,39]. For example, heart failure is associated with reduced cerebral perfusion, leading to ischemic changes that increase neuronal vulnerability. Inflammatory cytokines such as TNF-α and interleukin-1β play central roles in altering vascular permeability and neuronal function [40]. Moreover, recent studies highlight the bidirectional effects of the heart–brain axis, suggesting its role in neurological events such as seizures. Neuroinflammatory mediators, triggered by heart failure or other cardiac conditions, can influence both sympathetic activity and cerebral perfusion [41].

### 4.3. Radiomics

Radiomics represents a rapidly advancing field in medical imaging analysis, allowing for the extraction of high-dimensional, quantitative features from standard imaging data. In our study, elongation emerged as a significant radiomic parameter, correlating strongly with the risk of generalized seizures. Elongation quantifies the extent to which a lesion’s shape deviates from a perfect sphere, essentially capturing its structural deformation. Specifically, patients with elongation values above 1.45 were found to have a 7.78-fold increased likelihood of experiencing seizures, underscoring its clinical relevance.

**Figure 3:**
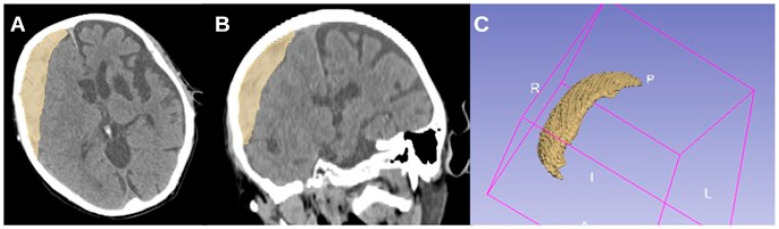
CT axial (**A**), coronal (**B**), and 3D reconstruction (**C**) of an acute subdural hematoma with high elongation (1.99615) in an 88-year-old male patient with generalized seizures.

**Figure 4:**
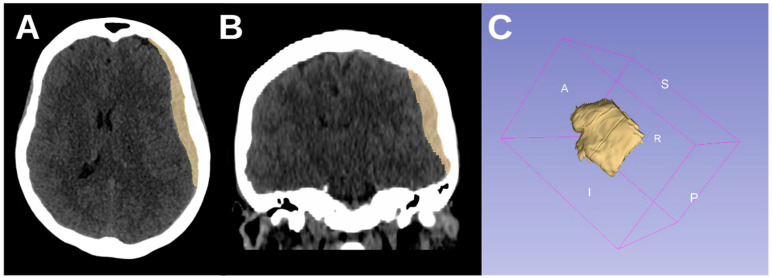
CT axial (**A**), coronal (**B**), and 3D reconstruction (**C**) of an acute subdural hematoma with lower elongation (1.38206) in a 68-year-old female patient with generalized seizures.

To illustrate this, Figure 3 and Figure 4 provide representative CT images of aSDH cases from our cohort with generalized seizures but different elongation values. Figure 3 shows a case with high elongation (1.99615), where the hematoma presents a markedly stretched, crescentic morphology with extensive cortical contact, aligning with our findings that higher elongation is associated with increased seizure risk. In contrast, Figure 4 depicts a case with lower elongation (1.38206).

These findings align with broader research on epilepsy, which has demonstrated the utility of radiomic features in predicting seizure outcomes across various neurological conditions, such as glioma-associated epilepsy (GAE) and epilepsy related to arteriovenous malformations (AVMs). The study by Gao et al. (2021) highlighted that radiomics-based models combining imaging features and clinical data achieved higher predictive accuracy for GAE than models relying on clinical data alone [15]. Similarly, Zhao et al. (2021) demonstrated the value of integrating radiomics and clinical parameters in predicting epilepsy in patients with unruptured AVMs, achieving an AUC of 0.82 in their combined model [16].

Feret diameter was also examined in our analysis and identified as a potential predictor in univariate assessments (*p* = 0.045). It represents the maximum distance between any two points along the hematoma’s perimeter, offering insights into its morphological characteristics. Although Feret diameter did not retain significance in multivariate models, it provides valuable information about the lesion’s size and structure, which may be relevant for future analyses.

Despite these advances, there remains a notable gap in the literature specifically addressing radiomic applications in subdural hematomas. Most existing studies focus on other neurological conditions, leaving a paucity of research on radiomics in the context of aSDH and its associated complications. This gap highlights the need for further studies to validate and expand upon our findings, particularly in diverse patient populations and across different imaging modalities.

While this study adds to the growing body of evidence supporting the role of radiomics in epilepsy prediction, it also underscores limitations in the existing literature. Future research should aim to standardize methodologies, explore multimodal imaging approaches, and validate radiomic models in larger, multicenter cohorts. These efforts will be essential for translating radiomics into practical tools for risk assessment and personalized care in patients with subdural hematomas.

### 4.4. Implications for Clinical Practice and Future Directions

The findings of this study have several important implications for clinical practice. Incorporating radiomic parameters like elongation into routine preoperative evaluations could enable personalized risk stratification, guiding decisions on prophylactic antiepileptic therapy or enhanced postoperative monitoring. Additionally, early identification of high-risk patients could inform resource allocation, particularly in intensive care units where monitoring capacities may be limited.

By incorporating elongation into routine preoperative assessments, clinicians could better identify patients at higher risk of postoperative seizures. For instance, patients with elongation values exceeding the identified threshold of 1.45 might benefit from early prophylactic antiepileptic therapy, reducing the likelihood of seizure onset during the critical postoperative period. Furthermore, elongation could guide decisions regarding the intensity of postoperative monitoring, ensuring that high-risk patients receive continuous electroencephalographic (EEG) surveillance or other neurocritical care interventions.

Future research should focus on multicenter validation studies to confirm the generalizability of these findings.

## 5. Limitations

Despite its contributions, this study has several limitations. First, the retrospective single-center design restricts the generalizability of our findings to broader populations. Additionally, the strict inclusion criteria, focusing solely on craniotomy patients, excluded those managed with alternative treatments, potentially introducing selection bias. The manual segmentation of hematomas, though carefully standardized, remains a source of variability and limits scalability. Moreover, while radiomic parameters such as elongation and Feret diameter were significantly associated with seizure risk, the small sample size may have reduced the power to detect associations for other variables. To further validate the adequacy of our sample size, we performed a power analysis for binary logistic regression using the Wald test. The required sample size for detecting an odds ratio of 7.778 with a power of 95% and a significance level of 5% was estimated at 58 patients. Lastly, the lack of long-term follow-up data precludes conclusions about the progression of post-traumatic epilepsy over time. Future multicenter prospective studies with automated segmentation tools and extended follow-up are warranted to validate these findings and ensure their applicability in diverse clinical settings.

## 6. Conclusions

This study highlights the significant potential of radiomic features in predicting generalized seizures in aSDH patients undergoing craniotomy. Among the parameters analyzed, elongation emerged as the most robust predictor, with patients exceeding the threshold value demonstrating a markedly higher seizure risk. Cardiac comorbidities and SOFA scores also contributed significantly to risk stratification, emphasizing the importance of integrating systemic and radiomic data for preoperative evaluations. These findings suggest that radiomics can complement existing clinical assessments, providing a foundation for personalized interventions aimed at reducing postoperative complications. By advancing preoperative risk stratification, this approach could improve outcomes and reduce the burden of post-traumatic epilepsy.

## Figures and Tables

**Table 3:** Binary logistic regression results.

Variable	Odds Ratio	95% CI	*p*-Value	Wald Statistic
Elongation	7.778	1.969–30.723	0.003	8.565
Feret diameter	2.851	0.853–9.515	0.090	2.873
SOFA	0.439	0.100–1.926	0.275	1.192

## Data Availability

The data presented in this study are available on request from the corresponding author due to the sensitive nature of clinical data and adherence to ethical guidelines.

## Abbreviations

The following abbreviations are used in this manuscript:

aSDH	acute subdural hematoma
AUC	area under the curve
AVMs	arteriovenous malformations
BBB	blood–brain barrier
CCL2	chemokine (C-C motif) ligand 2
CI	confidence interval
CNS	central nervous system
CT	computed tomography
DOACs	direct oral anticoagulants
EEG	electroencephalogram
GAE	glioma-associated epilepsy
ILAE	International League Against Epilepsy
OR	odds ratio
PTE	post-traumatic epilepsy
ROC	receiver operating characteristic
SD	standard deviation
SOFA	Sequential Organ Failure Assessment
TBIs	traumatic brain injuries
TNF-α	tumor necrosis factor-alpha
VIF	variance inflation factor
VKAs	vitamin K antagonists
